# Frequency of bone mineral density testing in adult kidney transplant recipients from Ontario, Canada: a population-based cohort study

**DOI:** 10.1186/s40697-016-0092-y

**Published:** 2016-01-16

**Authors:** Kyla L. Naylor, Guangyong Zou, William D. Leslie, Eric McArthur, Ngan N. Lam, Gregory A. Knoll, S. Joseph Kim, Lisa-Ann Fraser, Jonathan D. Adachi, Anthony B. Hodsman, Amit X. Garg

**Affiliations:** Institute for Clinical Evaluative Sciences (ICES), London, Ontario Canada; Institute of Health Policy, Management and Evaluation, University of Toronto, Toronto, Ontario Canada; Department of Epidemiology & Biostatistics, Western University, London, Ontario Canada; Department of Medicine, University of Manitoba, Winnipeg, Manitoba Canada; Division of Nephrology, University of Alberta, Edmonton, AB Canada; Division of Nephrology, Kidney Research Centre, Ottawa Hospital Research Institute, University of Ottawa, Ottawa, Ontario Canada; Division of Nephrology, University Health Network, University of Toronto, Toronto, Ontario Canada; Division of Endocrinology, Western University, London, Ontario Canada; Division of Rheumatology, McMaster University, Hamilton, Ontario Canada; Division of Nephrology, Western University, London, Ontario Canada; Institute for Clinical Evaluative Sciences, Room ELL-111, Westminster, London Health Sciences Centre, 800 Commissioners Road East,London, Ontario, N6A 4G5 Canada

**Keywords:** Bone mineral density, Kidney transplant recipient, Bone

## Abstract

**Background:**

We lack consensus on the clinical value, frequency, and timing of bone mineral density (BMD) testing in kidney transplant recipients. This study sought to determine practice patterns in BMD testing across kidney transplant centres in Ontario, Canada, and to compare the frequency of testing in kidney transplant recipients to non-transplant reference groups.

**Methods:**

Using healthcare databases from Ontario, Canada we conducted a population-based cohort study of adult kidney transplant recipients who received a transplant from 1994-2009. We used logistic regression to determine if there was a statistically significant difference across transplant centres in the decision to perform at least one BMD test after transplantation, adjusting for covariates that may influence a physician’s decision to order a BMD test. We used the McNemar’s test to compare the number of recipients who had at least one BMD test to non-transplant reference groups (matching on age, sex, and date of cohort entry).

**Results:**

In the first 3 years after transplant, 4821 kidney transplant recipients underwent 4802 BMD tests (median 1 test per recipient, range 0 to 6 tests), costing $600,000 (2014 CAD equivalent dollars). Across the six centres, the proportion of recipients receiving at least one BMD test varied widely (ranging from 15.6 to 92.1 %; *P* < 0.001). Over half (58 %) of the recipients received at least one BMD test post-transplant, a value higher than two non-transplant reference groups (general population with a previous non-vertebral fracture [hip, forearm, proximal humerus], 13.8 %; general population with no previous non-vertebral fracture, 8.5 %; *P* value <0.001 for each of the comparisons).

**Conclusions:**

There is substantial practice variability in BMD testing after transplant. New high-quality information is needed to inform the utility, optimal timing, and frequency of BMD testing in kidney transplant recipients.

**Electronic supplementary material:**

The online version of this article (doi:10.1186/s40697-016-0092-y) contains supplementary material, which is available to authorized users.

## ABRÉGÉ

**Mise en contexte:**

À ce jour, il n’existe aucun consensus sur la pertinence, au plan clinique, de demander une analyse de la densité minérale osseuse (DMO) chez les receveurs d’une greffe de rein, non plus que sur la fréquence ni le moment opportun pour y soumettre les patients après leur intervention.

**Objectifs de l’étude:**

L’étude avait pour but d’établir un schéma de pratique pour la mesure de la DMO dans plusieurs centres de transplantation rénale en Ontario, au Canada. On a également voulu comparer la fréquence de ces analyses chez les patients ayant reçu une greffe rénale par rapport à un groupe de référence constitué de patients non transplantés.

**Cadre et type d’étude:**

Il s’agit d’une étude rétrospective par cohorte représentative de la population, qui s’est tenue dans six centres de transplantation rénale en Ontario, au Canada.

**Patients:**

Il s’agit d’une cohorte de patients ayant reçu une greffe du rein entre 1994 et 2009.

**Mesures:**

Les renseignements sur la fréquence, le coût total et les variations dans le nombre d’analyses de la DMO pour une période couvrant les trois années suivant la greffe ont été compilés dans chacun des six centres. La fréquence des analyses de la DMO chez les patients greffés a été comparée à la fréquence de ces mêmes tests pratiqués chez les sujets de groupes témoins, apparentés sur les plans de l’âge, du sexe et de la date de leur admission dans la cohorte, mais n’ayant pas subi une greffe du rein.

**Méthode:**

L’analyse par régression logistique a été utilisée pour établir la présence de différences significatives du point de vue statistique entre les six centres de transplantation en regard de la décision d’effectuer au moins un test de DMO à la suite d’une greffe rénale. L’analyse a tenu compte des covariables qui pouvaient influencer les médecins traitants au moment de décider de procéder ou non à un test de DMO sur leurs patients greffés. Le test McNemar a été utilisé pour comparer le nombre de patients greffés ayant été soumis à une analyse de leur DMO par rapport au groupe témoin.

**Résultats:**

À l’intérieur d’une période de trois ans suivant leur transplantation, un total de 4802 analyses de DMO ont été demandées parmi les 4821 patients greffés du rein répertoriés dans les six centres participant à l’étude. La valeur médiane se situait à un test par patient sur une échelle allant de 0 à 6 tests par patient. Le coût total évalué pour ces 4802 analyses de DMO était de 600 000 CDN en 2014. La proportion de receveurs de greffe ayant été soumis à une analyse de leur DMO a fluctué considérablement d’un centre de transplantation à l’autre, avec des pourcentages variant de 15,6 % à 92,1 % (*P* < 0,001). Dans l’ensemble, on a analysé la DMO de plus de la moitié (58 %) des patients greffés au moins une fois après leur intervention. Ce résultat s’est avéré plus élevé que les pourcentages mesurés dans deux des groupes témoins non transplantés (valeur de *P* < 0,001 pour chaque cas) : un premier groupe constitué de gens qui avaient subi une fracture non vertébrale (hanche, avant-bras ou humérus proximal) par le passé (13,8 %) et un second groupe constitué de gens de la population générale n’ayant pas subi de fractures (8,5 %).

**Limites de l’étude:**

Les renseignements concernant les médicaments d’ordonnance administrés aux participants étaient incomplets et les valeurs de DMO étaient manquantes dans plusieurs cas. De plus, le faible taux de fractures subies par les participants ne permet pas d’établir une relation entre la valeur de DMO mesurée et le risque de fractures.

**Conclusions:**

Une variabilité importante a été constatée dans la pratique d’analyses de la DMO à la suite d’une transplantation rénale. Davantage de données sont nécessaires pour discuter de la pertinence d’effectuer ce test chez les receveurs de greffe rénale, ainsi que pour établir le moment opportun et la fréquence à laquelle les y soumettre après l’intervention.

## What was known before

Kidney transplant recipients have a higher risk of fracture compared to the healthy general population. However, the best way to identify recipients at high risk is unknown. Bone mineral density (BMD) is widely used in the general population to help identify patients with a high fracture risk, but its utility in the kidney transplant population is unclear. Limited evidence can lead to substantial practice variability. However, practice patterns for BMD testing in kidney transplant recipients from Ontario, Canada, are unknown.

## What this adds

Despite limited evidence on the utility of BMD testing, many kidney transplant recipients receive a test in the 3 years after transplant, and significant practice variability in BMD testing was observed across transplant centres. These results indicate future studies are needed to better understand the utility, frequency, timing, and cost-effectiveness of BMD testing in kidney transplant recipients.

## Background

It is well accepted that kidney transplant recipients have a higher risk of fracture compared to the healthy general population [1–4]. For example, Ramsey-Goldman et al.*,* found female kidney transplant recipients aged 25–44 years had an 18 times higher fracture risk compared to individuals from the general population of similar age and sex [1]; however, recent observations suggest that the absolute incidence is low with only 1.7 % of recipients sustaining a hip fracture in the 10-years after transplantation (high risk defined as a 10-year risk ≥3 %) [4–7]. The reasons for this higher risk are multifactorial and include pre-existing chronic kidney disease-mineral and bone disorder (CKD-MBD) and glucocorticoid administration after transplantation [8]. In the general population, Osteoporosis Canada guidelines recommend bone mineral density (BMD) testing is done in individuals at a high risk of fracture, as a decreased BMD can help risk stratify those individuals at higher risk of fracture [9–11]. However, in the kidney transplant population, the ability of BMD to predict fracture is unclear [12–14]. Limited evidence can lead to substantial practice variability. Therefore, we conducted a population-based study to determine the frequency, total cost, and the variability in BMD testing across all six transplant centres in Ontario, Canada. To help put the frequency of BMD testing into context, we also compared the frequency of testing in transplant recipients to non-transplant reference groups (matching on age, sex, and date of cohort entry).

## Methods

### Design and setting

We used linked healthcare databases from the province of Ontario, Canada to conduct this study. Universal access to physician and hospital services is provided to all Ontario residents. These datasets were linked using unique encoded identifiers and analyzed at the Institute for Clinical Evaluative Sciences (ICES). This study was approved by the institutional review board at Sunnybrook Health Sciences Centre, Toronto, Canada.

### Data sources

Information on Ontario kidney transplant recipients is provided by the Canadian Organ Replacement Register. Information on Ontario physicians’ billing claims for inpatient and outpatient services is reported by the Ontario Health Insurance Plan (OHIP). The Ontario Registered Persons Database provides information on demographics and vital status. Prescription drug utilization data is provided from the Ontario Drug Benefit Plan, which is a universal drug plan for individuals ≥65 years. It also provides information since April 1997 on special populations <65 years who are eligible for the program. The ICES Physician Database provides information on physician specialty. Emigration from the province was the only reason for loss to follow-up (0.5 % per year) [15].

### Primary cohort

#### Kidney transplant recipients

We included all adults (age ≥ 18 years) with a first kidney transplant from July 1, 1994 to December 31, 2009. We defined the date of the kidney transplant as the date of cohort entry (also referred to as the index date).

### Reference cohorts

To put the frequency of BMD testing into context, we matched recipients on age (±1 year), sex, and index date (±1 year) to two non-transplant reference cohorts; one group was considered to be at a low fracture risk where we would not expect frequent testing and one group was at an increased fracture risk where we would expect more frequent testing. Specifically, we matched recipients to the general population with no previous non-vertebral fracture (low fracture risk) (defined as proximal humerus, forearm, hip) and the general population with a previous non-vertebral fracture (increased fracture risk). When permitted by the available sample, we matched one recipient to four persons from the non-transplant reference cohort. Further detail on the cohort creation for these reference groups has been described elsewhere [4]. In Ontario, the 3-year incidence of fracture is higher in individuals with a previous non-vertebral fracture than in kidney transplant recipients (of which 1.6 % have a non-vertebral fracture [proximal humerus, forearm, hip] post-transplant) [4].

### Outcomes

We used physician fee-for-service billings to identify BMD by dual energy X-ray absorptiometry and, prior to April 1998, dual-photon absorptiometry tests (Additional file 1) [16]. In Ontario, these data are largely complete with approximately 94 % of physicians submitting such billing [17]. These BMD billing codes have been successfully used in several prior studies [18, 19]. We tabulated the number of BMD tests in the 3 years following kidney transplantation; multiple billings for a BMD test for a given person on the same day were counted as one test. We selected a 3-year follow-up to allow enough time for recipients to undergo multiple BMD tests; Osteoporosis Canada guidelines recommend a repeat BMD test in the 1–3 years after the initial test [9]. To calculate the total cost of the BMD tests, we included all associated billings (technical component of the test and professional component [e.g. physician interpreting the BMD test]) and accounted for inflation; additional information on billings can be found in Additional file 2.

### Statistical analysis

We used medians (interquartile range [IQR]) or means (standard deviation) to summarize baseline characteristics for continuous data and proportions to summarize categorical data. To compare baseline characteristics between recipients with at least one BMD test to those without a BMD test, we used the chi-square, Mann-Whitney *U* test, or Student’s *t* test as appropriate. We stratified the frequency of BMD testing by sex (men versus women) and age at the time of transplantation (<50 versus ≥50 years). We selected this age dichotomization for several reasons: kidney transplant recipients aged ≥50 years are at an increased fracture risk compared to younger recipients [4, 20]; favourable statistical properties (median age of our cohort was 50 years); and Osteoporosis Canada guidelines recommend BMD testing in individuals aged 50–64 years who have clinical risk factors for fracture (e.g. prolonged high-dose glucocorticoid use) [9]. We used logistic regression to determine if there was a statistically significant difference across transplant centres in the decision to perform at least one BMD test after transplantation. We adjusted for covariates that may influence a physician’s decision to order a BMD test (age, sex, previous non-vertebral fracture, and comorbidities [as measured by the Charlson comorbidity index [21]]). To determine if there were changes over time in the number of BMD tests performed, we used the Cochran-Armitage test for trend. To compare the number of recipients who had at least one BMD test to the matched non-transplant reference groups, we used McNemar’s test. We considered a two-sided *p* value <0.05 as statistically significant. We performed all analyses using the Statistical Analysis Software (SAS version 9.3).

## Results

### Baseline characteristics

We included 4821 kidney transplant recipients with a total observational time of 13,943 person-years; 304 (6.3 %) recipients died within 3 years. Comparing recipients who had at least one BMD (*n* = 2786) to recipients who did not (*n* = 2035), recipients with at least one BMD were significantly more likely to be women (42.4 versus 29.4 %; *P* < 0.001) and to have received a transplant in the later years of cohort entry (39.3 versus 25.5  %; *P* < 0.001); there was no significant difference in history of a previous non-vertebral fracture prior to transplant (2.4 versus 2.0 %) (Table 1). Matching characteristics were similar between recipients and the non-transplant reference groups (Additional file 3).

**Table 1:** Baseline characteristics of kidney transplant recipients classified by presence of at least one bone mineral density test in the 3 years after transplantation^a^

	Bone mineral density test		
Characteristic	Yes	No	*P* value
	(*n* = 2786)	(*n* = 2035)	
Age, years	50 (39–59)	49 (38-59)	0.04
Women	1182 (42 .4 %)	599 (29.4 %)	<0.001
Transplant era			<0.001
1994–1997	290 (10.4 %)	624 (30.7 %)	
1998–2001	631 (22.6 %)	480 (23.6 %)	
2002–2005	769 (27.6 %)	413 (20.3 %)	
2006–2009	1096 (39.3 %)	518 (25.5 %)	
Diabetes	690 (24.8 %)	565 (27.8 %)	0.02
Previous non-vertebral fracture	68 (2.4 %)	41 (2.0 %)	0.33
Charlson comorbidity index^b^	2.6 ± 1.0	2.7 ± 1.2	0.002

Data are median (interquartile range), mean (± SD) or numbers (percent)

Abbreviation: *SD* standard deviation

^a^Age and transplant era were assessed at the time of transplant (index date). Diabetes and Charlson comorbidity index were assessed in the 3 years prior to the transplant date. Prior non-vertebral fracture was defined as a composite of proximal humerus, forearm, hip fractures from 1991 to transplant date (index date)

^b^All recipients with a Charlson comorbidity index (CCI) of 0 were given a score of 2 and those with a score of 1 were given a score of 3; one of the variables in the CCI is presence of end-stage renal disease which automatically results in recipients receiving a score of 2

### Bone mineral density

Approximately 58 % (*n* = 2786) of kidney transplant recipients underwent at least one BMD test within 3 years of receiving their transplant and 22 % (*n* = 1047) of recipients underwent a BMD test in the 3 months following transplant. Among those with at least one BMD test, the median time after transplant to first BMD was 133 days (IQR 62–372 days). A total of 68.1 % of female recipients aged ≥50 years underwent a BMD test, a higher proportion than the other three age and sex strata (*P* < 0.005) (Table 2). There were a total of 4802 BMD tests (median 1, range 0–6 tests per recipient) and almost one third (31.7 %) of recipients underwent more than one BMD test in the 3 years after transplant (Additional file 4). The total cost of these tests was $614,997 (CAD 2014 equivalent dollars) (approximately $128 per recipient) across the 18-year study period. The proportion of recipients who underwent at least one BMD test in follow-up varied from 15.6 to 92.1 % (*P* < 0.001) across the six Ontario transplant centres. The variation across transplant centres persisted after adjustment for recipient age, sex, history of previous non-vertebral fracture, and comorbidities (logistic regression model, *P* < 0.001). When information on the ordering physician was available (96 % of tests), BMD tests were most commonly ordered by nephrologists (67.8 %) and family physicians (16.5 %), followed by general internists (5.0 %), rheumatologists (3.4 %), and endocrinologists (2.4 %).

**Table 2:** Number (proportion) of kidney transplant recipients with at least one bone mineral density test in the 3 years after transplantation by age and sex

	Kidney transplant recipients
	(*n* = 4821)
Overall	2786 (57.8 %)
Women <50 years	612 (64.8 %)
Women ≥50 years	570 (68.1 %)
Men <50 years	741 (50.7 %)
Men ≥50 years	863 (54.7 %)

### Non-transplant reference groups

**Fig. 1:**
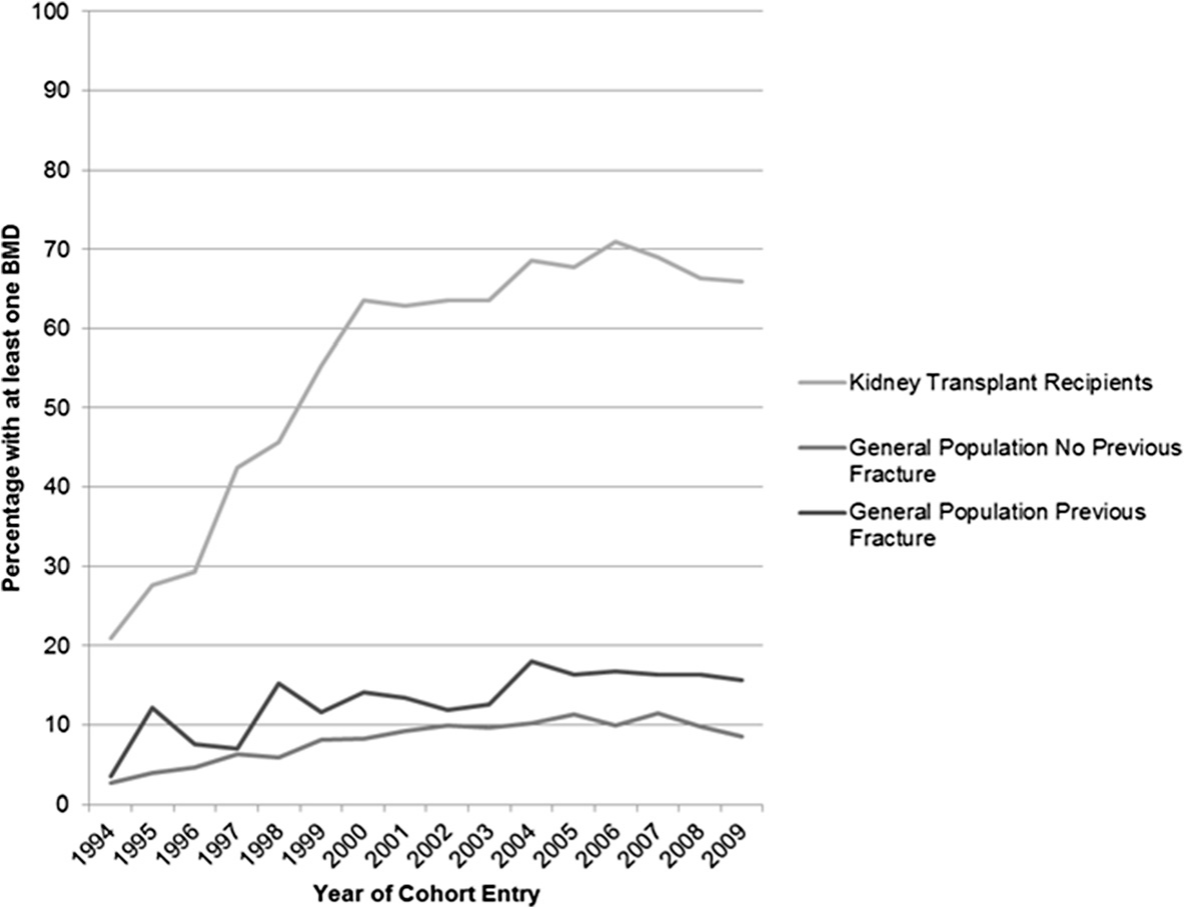
Kidney transplant recipients, individuals from the general population with a previous non-vertebral fracture and individuals from the general population with no previous non-vertebral fracture with at least one bone mineral density test in the 3 years after cohort entry, presented by year of cohort entry (*P* for trend <0.001 for all three cohorts)

In the general population with a previous non-vertebral fracture (*n* = 4821), there were 863 BMD tests (range 0–4) in the 3 years after the index date compared to 4802 BMD tests in the recipient population. In the general population with no previous non-vertebral fracture (*n* = 19,284), there were 1936 BMD tests (range 0–4). There were a significantly higher number of kidney transplant recipients with at least one BMD (58 %) in the 3-year follow-up versus both matched reference groups (13.8 % general population with a previous non-vertebral fracture and 8.5 % general population with no previous non-vertebral fracture, respectively, *P* < 0.001 for each paired comparison) (Table 3). Individuals who had an index date (cohort entry date) in more recent years were more likely to have underwent at least one BMD test in follow-up (recipients who transplanted in 1994, 20.9 and 66.4 % in 2009; general population with a previous non-vertebral fracture, 3.5 % in 1994 and 15.6 % in 2009; general population with no previous non-vertebral fracture, 2.6 % in 1994 and 8.5 % in 2009; *P* for trend <0.001) (Fig. 1).

**Table 3:** Number (proportion) of kidney transplant recipients with at least one bone mineral density test in the 3 years of follow-up compared to several reference groups matched on age, sex, and index date

Population	N (%)	*P* value*
Kidney transplant recipients (*n* = 4821)	2786 (57.8 %)	Reference
General population with no previous non-vertebral fracture (*n* = 19,284)	1645 (8.5 %)	<0.001
General population with a previous non-vertebral fracture (*n* = 4821)	665 (13.8 %)	<0.001

Matched on age (±1 year), sex, and index date (±1 year)

*Paired *P* value

### Bisphosphonates

Of the 3540 recipients who had prescription drug coverage through universal healthcare benefits, 646 (18.2 %) were prescribed bisphosphonates. Of recipients prescribed bisphosphonates, 548 (84.8 %) of these prescriptions were filled at a median of 57 days (IQR 21 to 175 days) after the BMD test, with 417 receiving a bisphosphonate prescription in the first 6 months after a BMD test.

## Discussion

In Ontario, Canada, we found that over half of the kidney transplant recipients underwent at least one BMD test in the subsequent 3 years after transplant, and many recipients underwent multiple tests. The frequency of BMD testing varied widely by centre—from as few as 15 % of recipients undergoing a BMD test to as many as 92 %, and this variability was not explained by recipient characteristics. Kidney transplant recipients were significantly more likely to undergo a BMD compared to two matched non-transplant reference groups. Our results suggest that BMD testing is commonly performed in kidney transplant recipients despite weak evidence in the literature supporting its widespread use.

The results of our population-based multicentre study extend the findings of two prior single centre reports with smaller sample sizes. In the first study of kidney transplant recipients (*n* = 326) from Manitoba, Canada, almost 60 % of recipients were found to have had at least two BMD tests within approximately 8 years of their transplant [22]. The second study from Akaberi et al. found that 670 BMD tests were performed in 238 kidney transplant recipients (75 % had at least two BMD tests) from Sweden over 12 years [12]. The centres in these two prior studies had protocols in place for routine BMD testing, and so the frequency of BMD testing would be expected to be high. In contrast, in our study, only a few of the transplant programs had a protocol for BMD testing (information provided by the six Ontario transplant centres, personal communication).

Particularly striking is the high number of kidney transplant recipients who underwent multiple BMD tests in the 3 years after transplantation, at a high cost to the healthcare system. For example, almost one third of kidney transplant recipients underwent two or more BMD tests within 3 years of their transplant; in the non-transplant population, the benefits of performing multiple BMD tests over several years have been questioned [23, 24], especially given the increasing knowledge of unwarranted screening harms [25, 26].

The benefit of BMD tests in kidney transplant recipients remains uncertain. First, the utility of BMD in predicting fracture in kidney transplant recipients is unclear [12–14]. For example, the Kidney Disease Improving Global Outcomes (KDIGO) guidelines for CKD-MBD suggest that patients with an estimated glomerular filtration rate >30 mL/min/1.73 m^2^ have their BMD assessed in the first 3 months after kidney transplant if they received glucocorticoids or have other risk factors for osteoporosis [8]. However, given the limited evidence, this suggestion was given the weakest grade of evidence [8]. It is important to note that this recommendation is currently being reassessed in the revised version of the guidelines [27] in light of recent evidence finding that BMD may be predictive of fracture in individuals with CKD, including dialysis [28, 29]; however, there is still conflicting evidence in kidney transplant recipients [12–14]. Second, given the high incidence of adynamic bone disease (i.e. low turnover) in kidney transplant recipients, the KDIGO guidelines suggest that a bone biopsy may be needed to guide treatment decisions; this limits the clinical usefulness of BMD testing post-transplant [8]. Third, and perhaps most relevant, recent research suggests in contrast to what has been previously reported, most kidney transplant recipients will not fracture and have an average mean BMD for age and sex [4, 5, 12, 22, 30]. Note, however, that the lower than expected fracture incidence and normal BMD may be the result of increased monitoring of bone health after transplant. Taken together, this suggests there may be little need to perform BMD tests routinely.

It is important to note that BMD testing may alter clinical practice. Many transplant recipients were prescribed a bisphosphonate in the first 6 months after receiving a BMD test. However, the efficacy of this and other fracture prevention strategies in kidney transplant recipients remains uncertain [31].

We make several recommendations based on the findings in this study. First, given the uncertainty that exists in the ability of BMD to predict fracture in kidney transplant recipients, new measures that have been found to predict fracture independent of BMD should be examined. For example, the trabecular bone score assesses bone quality (microarchitecture) and has been found to predict fracture in the general population [32–34]. This measure could be useful at predicting fracture in kidney transplant recipients given bone quality is particularly affected in recipients with CKD-MBD [8]. Second, new high-quality information from prospective observational studies and clinical trials is needed to guide the optimal recommended timing and frequency of BMD testing. Such studies should also assess the ability of BMD to predict fracture and its cost-effectiveness.

Our study has some limitations. First, we did not have drug dispensing information for the entire transplant cohort (only those who were covered by provincial drug benefits). While we were unable to characterize immunosuppression use at the patient level, during the time frame of this study, steroids were nearly universally prescribed at the Ontario transplant centres. Second, we only knew if a BMD was done, without information on the BMD value. However, the former supported the primary objective of this study—to determine the frequency of BMD testing in the first 3 years after transplant across several kidney transplant centres. Third, due to the low number of fracture events, we were not able to determine if transplant centres with more BMD tests had fewer fracture events. Moreover, the small number of recipients with a previous non-vertebral fracture may have limited our statistical power to determine whether recipients with a previous fracture were more likely to undergo a BMD test. Fourth, the variability in BMD testing we observed across transplant centres was in the setting of universal healthcare benefits. It is possible that these results may not generalize to other types of healthcare systems; variability across transplant centres might be even greater in jurisdictions without such healthcare benefits, as economic factors may also influence testing. Finally, we did not assess the impact of the KDIGO CKD-MBD guidelines on BMD testing. However, this guideline received the weakest grade of evidence; therefore, its uptake would likely be variable across transplant centres as demonstrated in this study.

## Conclusions

Many kidney transplant recipients underwent a BMD test in the 3 years after transplantation despite the lack of evidence to suggest BMD can accurately predict fracture. These results highlight the need for further studies to understand the utility, frequency, timing, and cost-effectiveness of BMD testing in kidney transplant recipients.

## Additional files

Additional file 1:
**Database codes for bone mineral density tests.** (PDF 83 kb) Additional file 2:
**Supporting methods.** (PDF 143 kb) Additional file 3:
**Baseline characteristics of reference groups.** (PDF 202 kb) Additional file 4:
**Frequency of bone mineral density tests performed in kidney transplant recipients.** (PDF 140 kb)

## Abbreviations

BMD: bone mineral density
CKD-MBD: chronic kidney disease-mineral and bone disorder
IQR: interquartile range
ICES: Institute for Clinical Evaluative Sciences
KDIGO: Kidney Disease Improving Global Outcomes
OHIP: Ontario Health Insurance Plan
