# Supplementary material for: Frequency of bone mineral density testing in adult kidney transplant recipients from Ontario, Canada: a population-based cohort study
Source: Can J Kidney Health Dis. 2016 Jan 16;3:2. doi: 10.1186/s40697-016-0092-y (PMC4715326; doi:10.1186/s40697-016-0092-y)
Supplement: Additional file 1: — Database codes for bone mineral density tests. (PDF 83 kb) [file 40697_2016_92_MOESM1_ESM.pdf]

Additional File 1: Database codes for bone mineral density tests

	OHIP Fee Codes
**Dual-photon absorptiometry**	J654 *Bone mineral density by single proton method*<br>J655 *Total boday calcium proton actiation*<br>J656 *Bone min. content dual-photon absorbtiomet. 2 or more sites*<br>J688 *Bone mineral content by dual photon single site*<br>J854 *Bone mineral density by single photon method*<br>J855 *Total body calcium - neutron activation*<br>J856 *Bone min. content dual-photon absorbtiomet. 2 or more sites*<br>J888 *Bone mineral content by dual photon absorb*
**Dual energy x-ray absorptiometry**	X145 *Bmd - baseline test, one site*<br>X146 *Bmd - baseline test, two or more sites*<br>X149 *Bone mineral density high risk 1 site*<br>X152 *Bone mineral density low risk 1 site*<br>X153 *Bone mineral density low risk 2+ sites*<br>X155 *Bone mineral density high risk 2+sites*<br>X157 *Diag. rad. bone density (mineral content) measurement*

Abbreviations: OHIP, Ontario Health Insurance Plan
