# Supplementary material for: Frequency of bone mineral density testing in adult kidney transplant recipients from Ontario, Canada: a population-based cohort study
Source: Can J Kidney Health Dis. 2016 Jan 16;3:2. doi: 10.1186/s40697-016-0092-y (PMC4715326; doi:10.1186/s40697-016-0092-y)
Supplement: Additional file 2: — Supporting methods. (PDF 143 kb) [file 40697_2016_92_MOESM2_ESM.pdf]

Additional File 2: Supporting Methods

Ontario Health Insurance Plan (OHIP) Billings

We included fee suffixes A, B, and C in the OHIP fee schedule. Fee suffix A was used prior to April 1, 2001 to describe both the technical and physical component of the exam. After April 1, 2001 fee suffixes B (technical component of the exam) and C (professional component) were required to be billed separately (1).

Reference

1. Ontario Ministry of Health and Long term Care. Schedule of Benefits. Ontario: Queen's Printer; 2014.
