# Supplementary material for: Frequency of bone mineral density testing in adult kidney transplant recipients from Ontario, Canada: a population-based cohort study
Source: Can J Kidney Health Dis. 2016 Jan 16;3:2. doi: 10.1186/s40697-016-0092-y (PMC4715326; doi:10.1186/s40697-016-0092-y)
Supplement: Additional file 3: — Baseline characteristics of reference groups. (PDF 202 kb) [file 40697_2016_92_MOESM3_ESM.pdf]

Additional File 3: Baseline characteristics of reference groups[¥]

Characteristic	**Kidney transplant recipients** (n=4821)	**General population with no previous non-vertebral fracture** (n=19,284)	**General population with a previous non-vertebral fracture** (n=4821)
Age, years	50 (38-59)	50 (38-59)	49 (38-59)
Women	1781 (36.9%)	7124 (36.9%)	1781 (36.9%)
**Era**			
1994-1997	914 (18.9%)	3655 (19.0%)	906 (18.8%)
1998-2001	1111 (23.1%)	4424 (22.9%)	1083 (22.4%)
2002-2005	1182 (24.5%)	4776 (24.8%)	1214 (25.2%)
2006-2009	1614 (33.5%)	6429 (33.3%)	1618 (33.6%)
Diabetes	1255 (26.0%)	1527 (7.9%)	503 (10.4%)
Prior non-vertebral fracture[‡]	109 (2.3%)		

Data are median (interquartile range) or numbers (percent)
[¥] Matched on age (±1 year), sex, and index date (±1 year)
[‡] Prior non-vertebral fracture defined as a composite of proximal humerus, forearm, hip fractures from 1991 to cohort entry.
Note: The reference group general population with no previous non-vertebral fracture has no previous fracture as this was a requirement to enter the cohort. The reference group general population with a previous non-vertebral fracture has 100% sustaining a fracture prior to cohort entry as this was a requirement for cohort entry.
