# Supplementary material for: Frequency of bone mineral density testing in adult kidney transplant recipients from Ontario, Canada: a population-based cohort study
Source: Can J Kidney Health Dis. 2016 Jan 16;3:2. doi: 10.1186/s40697-016-0092-y (PMC4715326; doi:10.1186/s40697-016-0092-y)
Supplement: Additional file 4: — Frequency of bone mineral density tests performed in kidney transplant recipients. (PDF 140 kb) [file 40697_2016_92_MOESM4_ESM.pdf]

Additional File 4: Frequency of bone mineral density tests performed in kidney transplant recipients

Number of BMD tests per recipient	N (%)
0	2035 (42.2%)
1	1259 (26.1%)
2	1081 (22.4%)
3	412 (8.5%)
4	27 (0.6%)
≥5	7 (0.1%)

Abbreviation: BMD, bone mineral density
